# Collapsing Glomerulopathy in a Child with Galloway-Mowat Syndrome

**DOI:** 10.1155/2016/4386291

**Published:** 2016-06-14

**Authors:** Cengiz Zeybek, Gokalp Basbozkurt, Salih Hamcan, Ayhan Ozcan, Davut Gul, Faysal Gok

**Affiliations:** ^1^Department of Pediatric Nephrology, Gulhane Military Medical Academy, Etlik, Kecioren, 06100 Ankara, Turkey; ^2^Department of Pediatrics, Gulhane Military Medical Academy, Etlik, Kecioren, 06100 Ankara, Turkey; ^3^Department of Radiology, Gulhane Military Medical Academy, Etlik, Kecioren, 06100 Ankara, Turkey; ^4^Department of Pathology, Gulhane Military Medical Academy, Etlik, Kecioren, 06100 Ankara, Turkey; ^5^Department of Medical Genetics, Gulhane Military Medical Academy, Etlik, Kecioren, 06100 Ankara, Turkey

## Abstract

Galloway-Mowat syndrome (GMS) is an autosomal recessive disorder with a poor prognosis that was first defined as a triad of central nervous system involvement, hiatal hernia, and nephrotic syndrome. However, this syndrome is now known to have a heterogeneous clinical presentation. The nephrotic syndrome is steroid resistant and is responsible for the outcome. The combination of collapsing glomerulopathy and GMS is very rare. A 26-month-old boy presented with steroid-resistant nephrotic syndrome associated with neurologic findings, including microcephaly, psychomotor retardation, and nystagmus. Magnetic resonance imaging showed marked cerebral atrophy, optic atrophy, and hypomyelination. A renal biopsy was consistent with collapsing glomerulopathy. If collapsing glomerulopathy is associated with neurological abnormalities, especially with microcephaly, clinicians should consider GMS as a possible underlying cause.

## 1. Introduction

Galloway-Mowat syndrome (GMS; MIM# 251300) is a rare autosomal recessive disease characterized by the combination of nephrotic syndrome (NS) and central nervous system involvement [1]. Since Galloway and Mowat first reported two siblings with early-onset NS, microcephaly, and hiatal hernia in 1968, more than 60 cases of GMS have been reported, with an expanding spectrum of phenotypic findings [2, 3]. Galloway-Mowat syndrome is a clinically heterogeneous disorder, and although included in the initial definition, hiatal hernia is no longer necessary for the diagnosis [2, 4–6]. Although some authors have tried to classify GMS according to the clinical presentation, no classification of the disease is currently accepted [7].

We report a boy with NS in whom a renal biopsy showed typical collapsing glomerulopathy associated with neurological findings consistent with GMS.

## 2. Case Presentation

**Figure 1:**
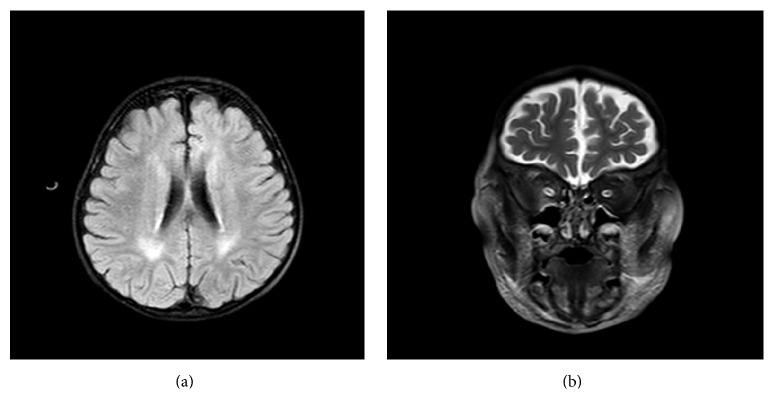
Brain magnetic resonance images: (a) The axial FLAIR image shows periventricular white matter signal changes compatible with hypomyelination. (b) Coronal T2-weighted magnetic resonance image shows bilateral optic atrophy and cerebral atrophy.

**Figure 2:**
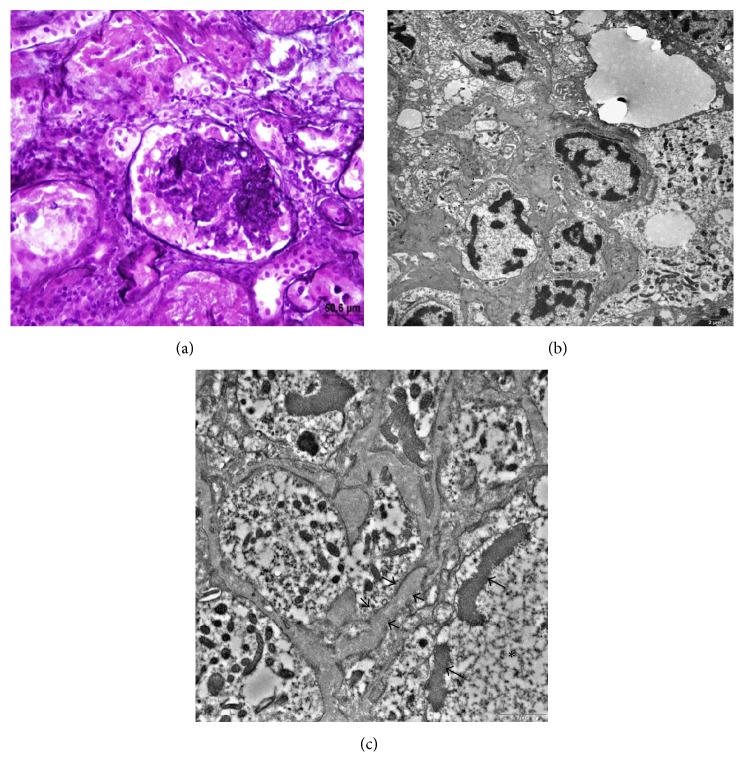
(a) Light microscopy shows wrinkling and collapse of the glomerular basement membranes accompanied by hypertrophied and hyperplastic podocytes forming pseudocrescents (Jones' methenamine silver stain, ×400). (b) Electron microscopy shows diffuse swelling in the endothelial layer and podocytes with electron-lucent transport vesicles (circle), diffuse foot process effacement, and wrinkling, irregular thinning, and thickening of the glomerular basement membranes (Electromicrograph, bar = 2 *µ*m). (c) At higher magnification, swollen podocytes with increased organelles, protein resorption droplets (long arrows), and a disrupted actin cytoskeleton (asterisk). The glomerular basement membranes had a radiolucent fibrillary appearance (short arrows), and the lamina densa had disappeared or was disrupted in many areas in the glomerular basement membrane (Electromicrograph, bar = 1 *µ*m).

A 26-month-old boy presented to our hospital with periorbital edema and ascites. The patient was being followed up at another center for nystagmus and microcephaly. He was the only child of a nonconsanguineous healthy couple. The family history was negative for renal diseases. The patient was a full-term product of an uncomplicated pregnancy with normal birth growth parameters (birth weight 3.25 kg, birth length 50 cm, and occipitofrontal circumference 35 cm). When the patient was brought to us, psychomotor retardation was apparent. He had been able to keep his head upright since being 6 months of age but could not walk or speak at presentation. When admitted to our hospital, his length was 85 cm (25th percentile), weight in an edematous state was 12.7 kg (11 kg without edema (10–25th percentile)), and the occipitofrontal circumference was 46 cm (below the 3rd percentile). The patient had hypertension (109/84 mmHg) and marked horizontal and vertical nystagmus. There was no facial dysmorphism. The abdominal examination showed gross ascites. The baseline investigations showed hemoglobin, 18.5 g/dL; total leukocyte count, 9600 cells/mm^3^; platelet count, 474 × 10^3^/*µ*L; serum urea, 86 mg/dL; serum creatinine, 0.7 mg/dL; low serum albumin (1.4 g/dL) and serum total protein (3.6 g/dL); and high serum triglyceride (1058 mg/dL), serum cholesterol (482 mg/dL), and 24-hour proteinuria (55 mg/m^2^/h). Viral markers (HBsAg, anti-HCV, HIV, EBV, CMV, and parvovirus) were negative; serum complement and antinuclear antibodies were normal. Microscopic hematuria (3+) and albuminuria (4+) were detected. Ultrasound showed normal-sized kidneys with increased cortical echotexture and loss of corticomedullary differentiation. With a diagnosis of NS, we administered 2 mg/kg/day prednisolone to the patient for 4 weeks with no reduction in proteinuria. Coenzyme Q10 was also administered because of the psychomotor retardation. The urine organic acid screening and tandem mass spectrometry results were normal. Brain magnetic resonance imaging revealed cerebral atrophy, bilateral hypomyelination in the periventricular white matter, and optic atrophy (Figure 1). The metabolic peak was not available on brain magnetic resonance spectroscopy. A renal biopsy was performed after the patient did not respond to 4 weeks of steroid treatment, and the treatment shifted to cyclosporine. The kidney biopsy showed 11 glomeruli and revealed that the glomerular capillary lumens were occluded by global wrinkling and collapse of the glomerular basement membranes with hypertrophy and hyperplasia of the overlying podocytes forming pseudocrescents, without inflammatory cells and Bowman capsule rupture (Figure 2(a)). Hypertrophied and hyperplastic podocytes contained numerous intracytoplasmic vesicles and protein resorption droplets. In addition, focal mild tubular degeneration, regeneration, and dilatation were observed, with mild to moderate interstitial inflammation. Some tubules contained red resorption droplets. Immunofluorescent examination revealed no deposits of IgG, IgA, IgM, C3, C1q, fibrin, or kappa and lambda light chain antibodies. On electron microscopic examination, semithin sections stained with toluidine blue revealed collapse of the glomerular basement membranes, with hypertrophy and hyperplasia of the overlying podocytes, forming pseudocrescents. Thin sections stained with lead citrate and uranyl acetate revealed wrinkling, irregular thinning, and thickening of the glomerular basement membranes, accompanied by diffuse foot process effacement of markedly hypertrophied and swollen podocytes with electron-lucent transport vesicles, increased organelles, and a disrupted actin cytoskeleton (Figures 2(b) and 2(c)). The glomerular basement membranes had a radiolucent fibrillary appearance, and the lamina densa, which consists mainly of collagen type IV and laminin, had disappeared or was disrupted in many areas in the glomerular basement membrane (Figure 2(c)). No electron-dense deposits or tubuloreticular inclusion bodies were observed in the glomeruli. These findings supported a diagnosis of collapsing glomerulopathy.

Genetic testing for the podocin mutation analysis revealed no mutation. We did not achieve clinical or laboratory improvement with cyclosporin therapy. We started peritoneal dialysis because the serum urea level increased to 288 mg/dL and the serum creatinine level to 3.19 mg/dL. However, after 2 months in the hospital, the patient died of* Candida* septicemia despite appropriate supportive care and antimycotic treatment. Next-generation DNA sequencing found no evidence of the WDR73 mutation.

## 3. Discussion

The kidney involvement in GMS comprises a broad spectrum ranging from mild nonnephrotic proteinuria to steroid-resistant NS with rapid progression to end-stage renal disease [8, 9]. Nephrotic syndrome often develops within the first months of life, at an average of 3 months, although later onset during childhood (44–198 months) has been reported [5, 10–12]. If the renal findings occur at less than 3 months of age, the brain formation and migration anomalies become more severe, and the patient usually dies early. If the renal findings occur later, both the renal disease and brain development anomalies are less severe [7, 13]. Pathologically, the renal involvement in GMS can be similar to calcineurin inhibitor toxicity, with striped fibrosis and tubular atrophy; other renal lesions include mesangial proliferation, microcystic dysplasia, minimal change NS, diffuse mesangial sclerosis, and focal segmental glomerulosclerosis [2–4, 9, 10, 14–16]. Altered renal histology has been reported in the same patient [15]. There is no typical age-specific renal histological pattern regardless of whether the renal failure is seen at early or older ages. Only two cases of collapsing glomerulopathy have been reported in GMS [8, 17]. Severe collapsing glomerulopathy was present in both patients and progressed rapidly to death. Our patient is the third reported GMS patient with collapsing glomerulopathy, and he died 2 months after developing NS.

Lin et al. claimed that although there is no specific electron microscopic lesion in GMS, foot process effacement along with irregular thickening of the basement membrane is pathognomonic [14]. However, this finding has not been discussed in subsequent studies [17]. In our case, these two findings were present (Figure 2(b)). Another important feature of GMS nephropathology is tubular atrophy associated with tubulointerstitial inflammation; this was present to a mild to moderate degree in our case [17]. In GMS, the most frequently reported vascular change is arteriolar medial hypertrophy [14, 18]. However, our patient, like most GMS cases, had normal arteries.

Neurological findings are universal in children with GMS and often precede renal abnormalities. The consistent morphological hallmark of the disease is microcephaly, which is often present at birth but can develop postnatally, as in our patient. In addition to microcephaly, severe developmental delay and structural brain abnormalities such as malformations of cortical development, hypomyelination, nystagmus, and cerebellar atrophy may occur in some patients [5, 19]. Severe, intractable seizures, not mentioned in early reports, have been increasingly recognized, being involved in more than half of recently reported cases [3, 9, 13]. Our patient did not have epilepsy but had cortical (especially frontal) atrophy, widespread hypomyelination, and optic atrophy.

Although no facial dysmorphism is seen in some patients, others may exhibit dysmorphic features such as a sloping narrow forehead, low-set large floppy ears, a high palate, abnormally shaped skull, micrognathia, arachnodactyly, and coarse hair [3, 9, 14, 18, 20]. Our patient did not have a dysmorphic appearance.

The molecular basis of GMS is currently unknown. In 1994, Cohen and Turner examined kidney tissues of the patients using antibodies against glomerular basal membrane, tubular basal membrane, type IV collagen, and laminin along with different stains in an attempt to understand the pathophysiology of the syndrome but did not find any abnormality [18]. In 2001, Srivastava et al. studied mutations of the synaptopodin, GLEEP1, and nephrin genes, which are expressed in both neurons and podocytes but did not find any mutations of these genes [21]. An analysis of candidate genes encoding proteins of the glomerular basal membrane (LAMB2 and LAMA5) and podocyte proteins (ITGB1, ITGA3, and ACTN4) failed to detect any causative mutation in GMS patients [6]. Very recently, however, WDR73 deficiency was identified in five individuals with GMS presenting with childhood-onset NS, postnatal microcephaly, severe intellectual disability, and homogenous brain magnetic resonance imaging features including cerebellar atrophy [8, 22]. WDR73, which is present in brain and kidney tissue, encodes a WD40-repeat-containing protein and plays a role in cell survival and the organization of neuronal and axonal microtubule regulation [8, 23]. Vodopiutz et al. screened the isolated cerebellar abnormalities and varying degrees of brain atrophy patients and clinically diagnosed GMS patients for the WDR73 mutation but detected only two mutations in 40 GMS patients (5%) [1]. They concluded that the WDR73 mutation is much more common in cerebellar atrophy associated with neurodegenerative diseases and this mutation may not always be associated with renal involvement. Moreover, glomerulopathy associated with the WDR73 mutation occurs in later years (5–12 years of age) [1, 8, 22]. We did not detect any WDR73 mutations in our case. It is likely that GMS is genetically heterogeneous, given the clinical heterogeneity of the syndrome.

As a result, our case is the third reported collapsing glomerulopathy case with GMS, and our patient died rapidly. Galloway-Mowat syndrome should be considered in collapsing glomerulopathy patients with neurological findings, especially with microcephaly.
